# A New Eye Dual-readout Method for MiRNA Detection based on Dissolution of Gold nanoparticles via LSPR by CdTe QDs Photoinduction

**DOI:** 10.1038/s41598-019-41898-4

**Published:** 2019-04-01

**Authors:** Yasaman-Sadat Borghei, Morteza Hosseini

**Affiliations:** 1 Department of Life Science Engineering, Faculty of New Sciences & Technologies, University of Tehran, Tehran, Iran; 2 Department of Pharmaceutical Biomaterials and Medicinal Biomaterials Research Center, Faculty of Pharmacy, Tehran University of Medical Sciences, Tehran, Iran

## Abstract

Breast cancer (BC) is the most frequent cancer that affects one in eight women worldwide. Recent advances in early cancer diagnosis anticipates more efficient treatment and prolong patient survival. MicroRNAs expression profiling plays a key role in diagnosis of cancer such as BC in early stages. For the first time we describe direct injection of hot electrons from plasmonic gold nanoparticles (AuNPs) to adsorbed water molecules with photoinduction of CdTe quantum dots (QDs) with emission wavelength at ~560 nm. As a result of hot electrons exiting from AuNPs with red color, gold cations (holes) are gradually discharged (AuNPs dissolution) leading to a colorless solution. Our group applied this phenomenon to propose a spectral method for miRNA recognition based on different responsive disaggregation and aggregation of CdTe QDs interacted with single strand DNA probes and DNA/RNA heteroduplex respectively resulting in a detection limit of 4.4 pM. This method has been applied also for the determination of miR-155 in the human breast carcinoma MCF-7 cells and normal human embryonic kidney cell line (HEK 293).

## Introduction

Since the discovery of microRNAs (miRNAs) in 1993, they have received extensive interest in biology and clinical research, including the diagnosis and treatment of a variety of diseases such as cancer^1–4^. Patterns of miRNA expression studies on breast cancer (BC) were first described in 2005^5,6^. BC types can be categorized based on miRNA expression profiling on the basis of their heterogeneity^7^. Regarding BC, it has been well known that the dysregulated miRNAs play significant roles in apoptosis, angiogenesis, invasion and metastasis^8^. Recently, some miRNA expression pattern have been described to be involved in breast cancer, including miR155. Human oncogenic miR-155 (oncomiR) has been described to be involved in the pathogenesis and progression of BC and the overexpression of miR-155 can render breast cancer cells resistant to chemotherapeutic agents^9–12^. It was reported that miR-155 downregulates SOCS1 in breast cancer, in turn leading to persistent activation of STAT3 signaling^13^. This activation results in inflammatory cascades and indicates the communicative role of miR-155 between inflammation and cancer^13^. In breast cancer tissue, the overexpression of miR-155 was observed and suggested as a risk factor for breast cancer^14^.

Thus, the diagnosis of their expression level has been suggested as a diagnostic and prognostic biomarker for early detection of breast cancer. Traditional methods for miRNA detection, including qRT-PCR, next-generation sequencing (NGS) and microarray have some strengths and weaknesses. For example, although qRT-PCR is the fastest among the three methods, yielding results in approximately 6 hours, the requirement of RNA and costs are relatively high compared to microarray and NGS. Moreover, it requires primers designed against existing sequences^15,16^. Therefore, achieving a cheaper, faster method without the need for special equipment is very valuable.

Today, gold nanoparticles (AuNPs)-based localized surface plasmon resonance (LSPR) as an optical sensor has been widely employed in various fields including disease diagnosis. This is due to its advantages such as sensitivity, simplicity, rapidity, being label-free, low-cost instrument and requirement of low sample volume. Because of their intrinsic interband transitions, these plasmonic AuNPs have strong absorption on the high energy side of their plasmon resonances^17,18^. Among numerous metal NPs, AuNPs and silver NPs have a unique LSPR feature which originates from the collective oscillations of electrons in conduction band after the interaction of the nanoparticle with UV-visible radiations^19^. When the light beam is irradiated, metal nanoparticles absorb a part of the photon and scatter another part. Thus, optical spectroscopy is the simplest technique to detect the LSPR on metal nanoparticles and is commonly based on extinction (absorption) or scattering assessments. Absorption is often employed to characterize systems containing nanoparticle colloids^20,21^.

**Figure 1:**
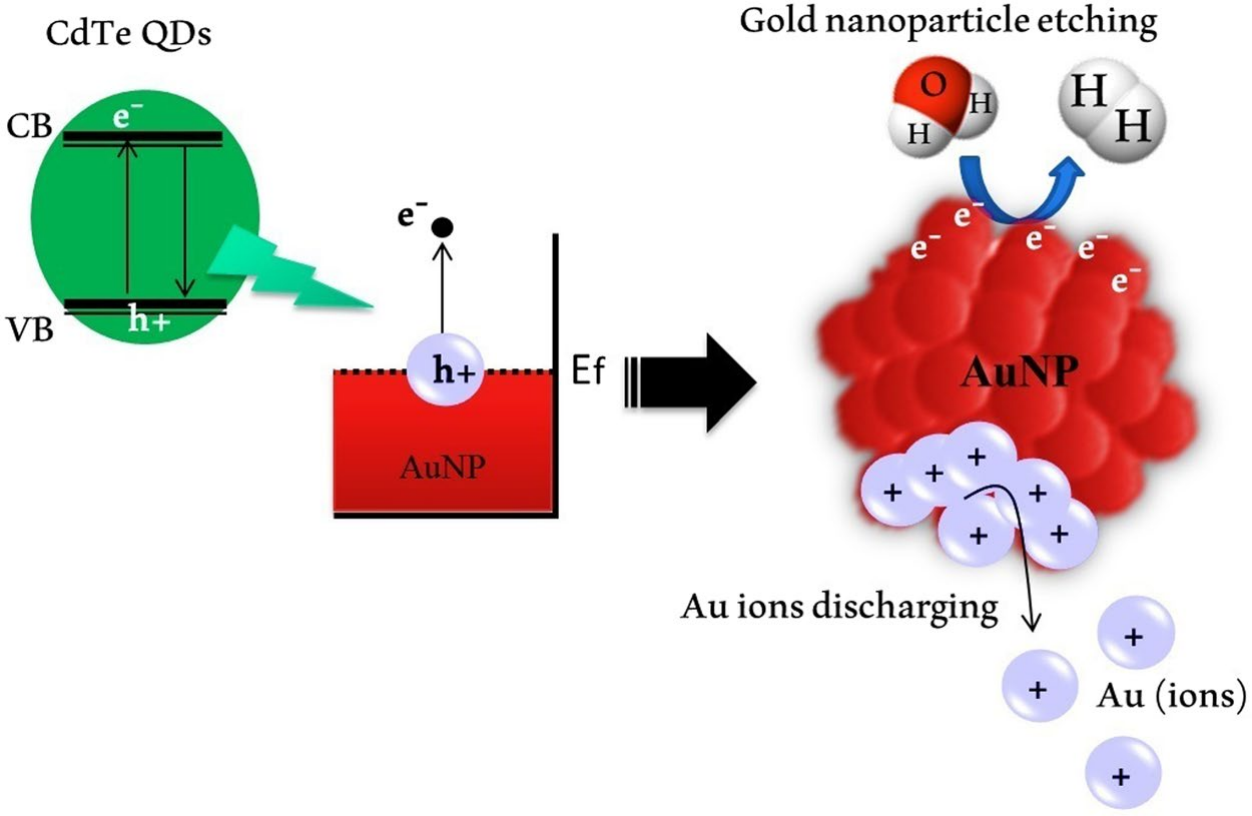
Mechanism of dissolution plasmonic AuNPs with water splitting via hot-electron injection by using CdTe QDs photoinduction.

For the first time, this technique has been used by *Robatjazi et al*.^22^ in solar-to-chemical energy conversion. Here for the first time our group has extended this mechanism for miRNA diagnosis. The LSPR-based miRNA detection was conducted through electron charging and discharging of Au cations (AuNPs dissolution)^23–28^ via electron transfer from semiconductor CdTe QDs to AuNPs under UV light irradiation, in the absence of miRNA (Fig. 1). The excitation of surface plasmonic AuNPs leads to the generation of hot electrons in the plasmonic AuNPs, which can be transferred to hydronium ion in water^22,29,30^. In the presence of specific miRNAs, synthetic DNA can be hybridized to them through the Watson–Crick base pairing forming a heteroduplex. Existence of the DNA/miRNA duplex leads to rapid aggregation of CdTe QDs followed by severe quenching of fluorescent intensity. Thus, in the presence of DNA/miRNA duplex LSPR of AuNPs inhibited and the LSPR band remained constant.

## Experimental

### Apparatus

Fluorescence analysis was performed utilizing a Perkin Elmer LS-55 fluorescence spectrometer (Buckinghamshire, UK). A xenon lamp was used as the source of excitation with monochromators spectral band widths of 10 nm for both emission and excitation. The size and shape of AuNPs and CdTe QDs were evaluated through Transmission Electron Microscopy (TEM) (Zeiss, EM10C, 80 KV, Germany). UV–vis spectra were recorded using a Specord 250 spectrophotometer (Analytik Jena, Germany).

### Materials and reagents

Dulbecco’s modified eagle medium (DMEM), fetal bovine serum (FBS) and penicillin/streptomycin were bought from Gibco (USA). Synthesis and purification of oligonucleotides was carried out by Pishgam Biotech Co (Tehran, Iran). The sequences are listed in Table S1. Purification of all DNA samples was done by PAGE and they were solvated in TE buffer (1 M Tris-HCl, 0.5 M EDTA). Thioglycolic acid (TGA), Cd(NO_3_)_2_, tellurium powder, and sodium borohydride (NaBH_4_) were acquired from Merck. Cell Culture Lysis Reagent (CCLR), 5X was bought from Sigma Aldrich. Other commercially available substances were purchased from Aldrich, Merck and Acros and used with no additional purification. Analytical reagent grade of all other reagents and ultrapure water (Milli-Q plus, Millipore Inc., Bedford, MA) was utilized through experiments.

The MCF-7 (human breast cancer cell line) and HEK 293 (from normal human embryonic kidney cell line) cell lines were used in the present study.

### Preparation of TGA capped-CdTe QDs

Preparation of QDs was carried out according to previously reported studies^31,32^. Briefly, 0.4 mmol Cd and 1.4 mmol thioglycolic acid (TGA) were mixed in 80 mL distilled water. The pH of this mixture was fixed to 10.0 by addition of NaOH. Afterwards, 0.8 mmol sodium borohydrate and Te powder were mixed in 10 mL distilled water in a flask, under forceful stirring and argon flow. Next, it was heated to 80 °C until a clear red solution of NaHTe was obtained. Cd-TGA mixture was heated at 100 °C under argon flow in a 250 mL three-neck flask. Next, 4.0 mL fresh NaHTe solution was injected into the flask. Finally, the mixture was refluxed at 100 °C for 2 hours.

### Synthesis of gold nanoparticles

50 mL aqueous solution (1 mM) of HAuCl_4_.3H_2_O in a flask with a reflux condenser was heated to boiling temperature under stirring. 10 mL trisodium citrate (38.8 mM) was then added to the mixture rapidly. The solution was kept in boiling status for 10 minutes while the color of the solution was changing slowly from yellow to red. After stopping the heating, the stirring was continued until the mixture was cooled down to room temperature^33^. The AuNPs solution was stored at 4 °C. The TEM imaging was used to determine the diameter and dispersion state of the prepared AuNPs. Based on the Beer’s law and by using the extinction coefficient (2.7 × 10^8^ M^−1^ cm^−1^) at 520 nm^34^, the concentration of the AuNPs solution was calculated to be about 4.4 nM. The diameter of the prepared AuNPs was in the range of 20–30 nm.

### Cell culture

MCF-7 cells and HEK 293 cells were cultivated in 25 cm^2^ tissue culture flasks (SPL, Korea) with 5 mL Dulbecco’s modified Eagle’s medium (Sigma, UK). The medium contains 10% heat inactivated FBS (Gibco) and 100 U/ml penicillin (Sigma, UK). The nurture of cell lines was carried out in a humidified atmosphere of 5% CO_2_ and 95% air at 37 °C for five days until a confluent cell monolayer was achieved. Growth medium was being substituted with fresh medium every 2 days or when needed (understood by a color alteration). This color alteration was originated from production of lactic acid and CO_2_ that lowered the pH level. After achieving the minimum confluency of 80%, cells were subjected to washing with phosphate buffered saline (PBS) and trypsinization for 10 min at 37 °C with 0.05% trypsin.

### Extraction of total RNA

Extraction of RNA was carried out using Cell Culture Lysis Reagent (CCLR) to disrupt MCF-7 and HEK 293 cells and then total RNA was extracted. Around 2.0 × 10^6^ cells were collected via centrifuging at 1000 rpm for 10 min. Culture medium was cautiously separated followed by washing the pellet twice with PBS. PBS was cautiously separated and 600 µL CCLR buffer was inserted. Cells were softly resuspended in CCLR buffer using vortex and then were incubated for 20 min. Then, 0.2 mL chloroform was added followed by a 20 s violent vortex. Subsequently, the mixture was centrifuged at 13,000 rpm for 20 min at 4 °C. Equal amount of isopropyl alcohol was added to the upper water. Then it was taken out and mixed evenly in −20 °C precipitation overnight. Later, another round of centrifugation was performed (20 min at 13,000 rpm at 4 °C). Next, supernatant was separated and removed in which, the pellet was washed by 80% ethanol with DEPC water and centrifuged for 20 min at 13,000 rpm at 4 °C. The ethanol was volatilized (tube dried) and the purified RNA was solvated in a suitable amount of DEPC water.

### Procedure

To determine the effects of hybridization of miRNA targets on LSPR band, various concentrations of miR-155 targets (10–100 pM) were added to 10 μL of DNA probe (100 pM) in 40 μL phosphate buffer (20 mM, pH = 6.5). Then the mixtures were heated to 90 °C for 10 min (denaturing step). The hybridization was performed by incubating at 37 °C for 1 h (annealing step). After that, 10 μL of QDs (1.1 nM) was added to above solutions. Then by the addition of AuNPs (40 μL 4.4 nM) into all of the samples, LSPR bands were measured. All experiments were completed at room temperature.

## Results and Discussion

### The Characterization of CdTe QDs and AuNPs

For LSPR-based detection, there needs to be an overlap between the absorption spectrum of AuNPs and fluorescence spectrum of QDs. Figure S1a shows the absorption spectra of both AuNPs and CdTe QDs and the fluorescence spectra of QDs. For AuNPs with a diameter of around 20–30 nm, the distinctive absorption peak at about 530 nm can be seen (Fig. S1c). CdTe QDs with a diameter of 2–3 nm (Fig. S1b) have the emission peak at 560 nm, which is coupled with the absorption peak of AuNPs. These spectral characteristics made it possible for AuNPs to be the absorber and CdTe QDs to be the emitter in the QDs–AuNPs system.

### Photoinduced Dissolution of AuNPs

**Figure 2:**
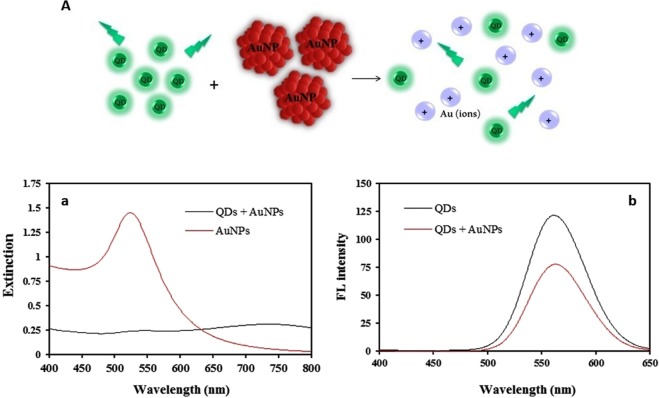
(**A**) Schematic representation of the dissolution plasmonic AuNPs after adding bare green CdTe QDs, and their spectra: (a) extinction band of “AuNPs” and “AuNPs + QDs”, and (b) the fluorescent behavior of bare green “QDs” and “QDs + AuNPs”.

It has been more than 50 years since the first study of the sophisticated process of dissolution of gold nanoparticles. This process was microscopic-modeled by *Cherevko et al*.^24^ Here, we observed large AuNPs dissolution due to direct hot electron transition from plasmonic AuNPs to water molecules upon plasmon decay. The fundamental elements of our system are (i) hot-carrier separation from AuNPs by CdTe QDs photoinduction, (ii) efficient hot-carrier generation, and (iii) water molecules which act as the absorber and are split to H_2_ (Fig. 1). The result of these events is the reduction in the extinction band intensity at 530 nm, and the color change of the reaction solution from red to the colorless (Fig. 2).

**Figure 3:**
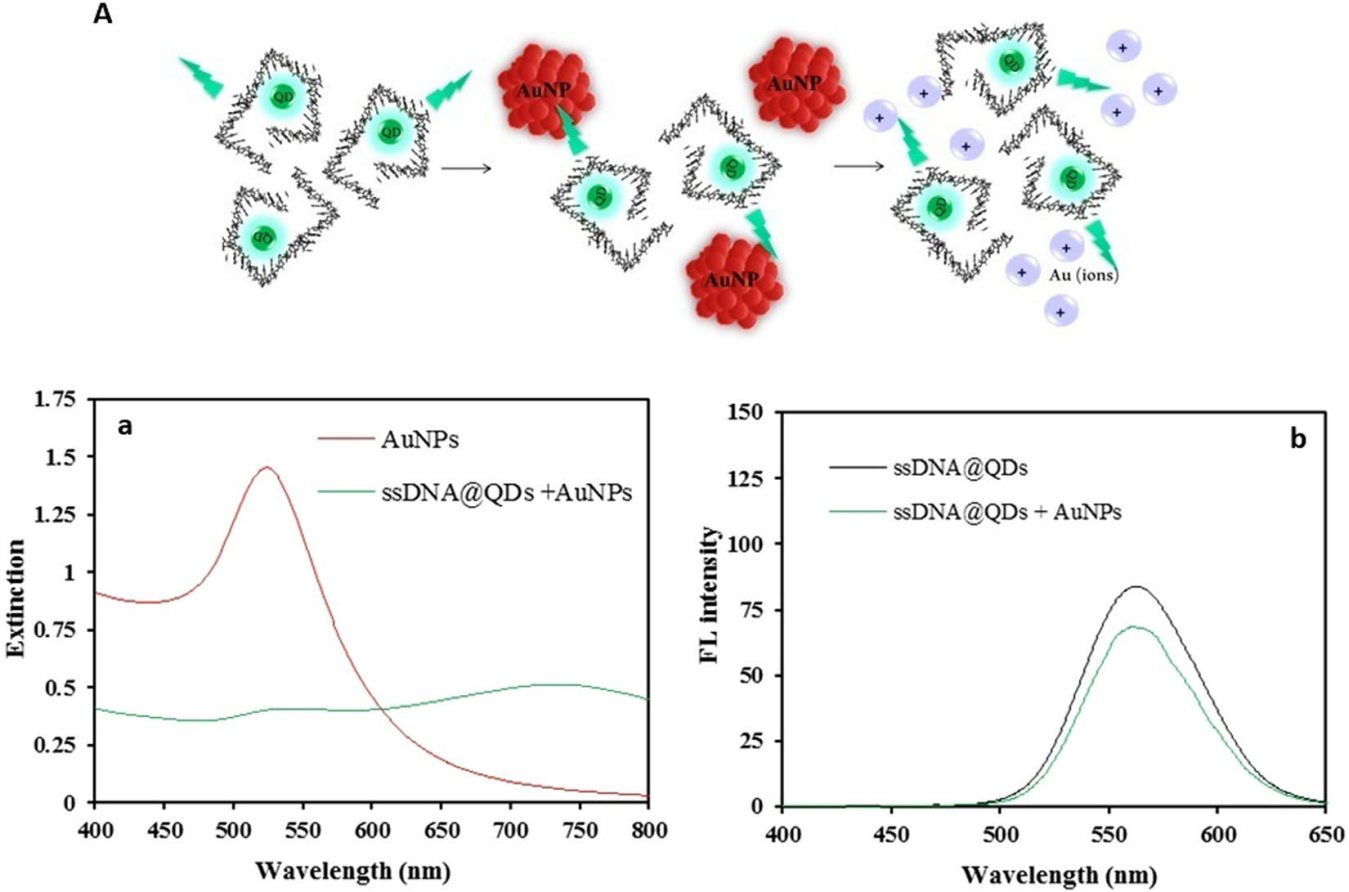
(**A**) Schematic representation of the dissolution plasmonic AuNPs after adding ssDNA probe@CdTe QDs. And their spectra (a) extinction band of “ssDNA@QDs + AuNPs”, and (b) the fluorescent behavior of “ssDNA@QDs” and “ssDNA@QDs + AuNPs”.

In the presence of the single-stranded DNA probe, the intensity of fluorescence emission of QDs does not change significantly and we have observed a decrease in the extinction band (Fig. 3).

**Figure 4:**
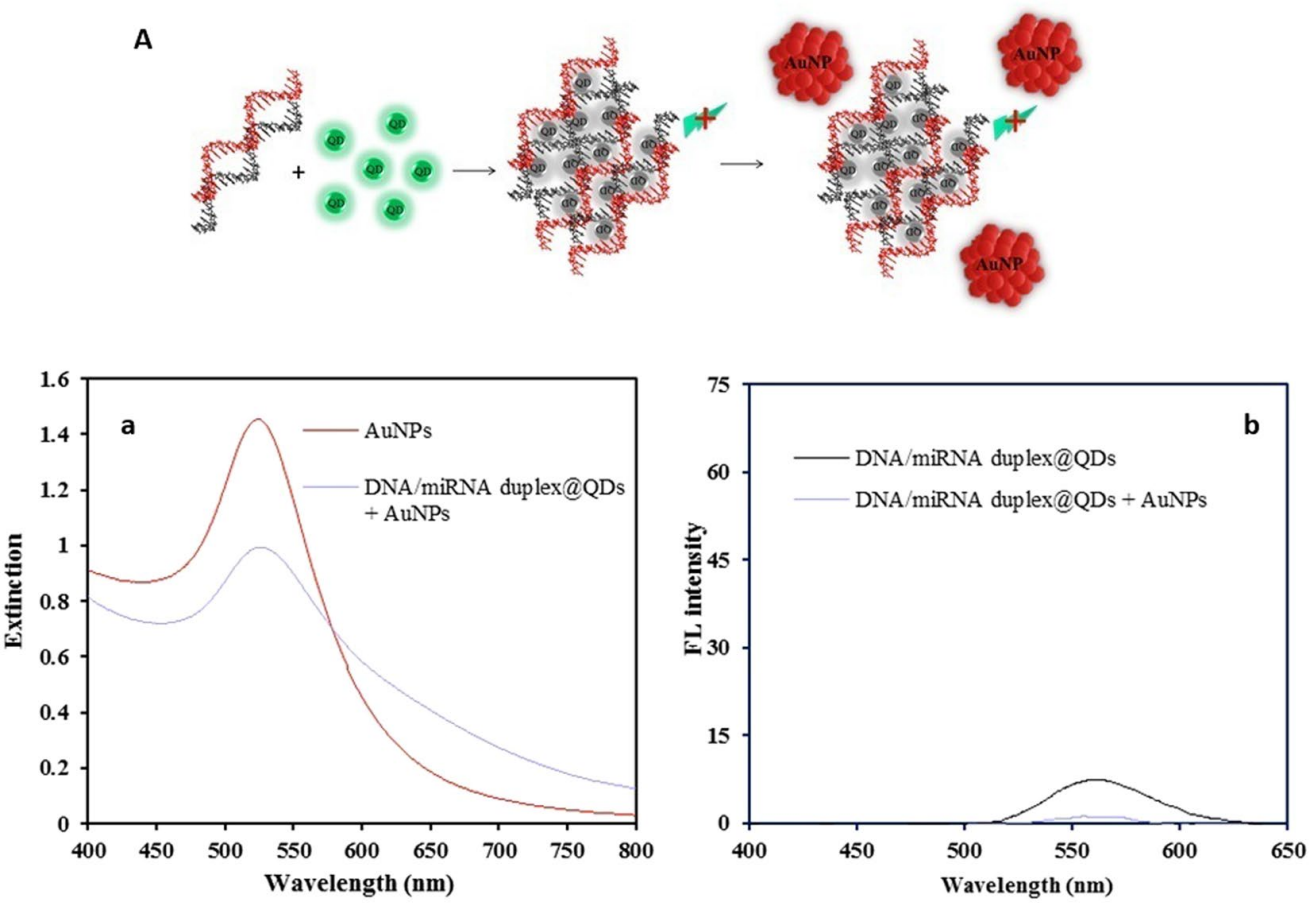
(**A**) Schematic representation of the stability of plasmonic AuNPs after adding dsDNA/miR-155@CdTe QDs, and their spectra: (a) extinction band of “dsDNA/miR-155@CdTe QDs + AuNPs”, and (b) the fluorescent behavior of “dsDNA/miR-155@QDs” and “dsDNA/miR-155@QDs + AuNPs”.

However, the usage of this technique in miRNA diagnosis is based on the aggregation of QDs and their quenching in the presence of the DNA/miRNA duplex. Therefore, with quantum dot fluorescence quenching, the plasmon of AuNPs are not excited and what is observed is the high-intensity extinction band at 530 nm (Fig. 4).

### Transmission electron microscopy analysis of AuNPs dissolution and CdTe QDs aggregation

**Figure 5:**
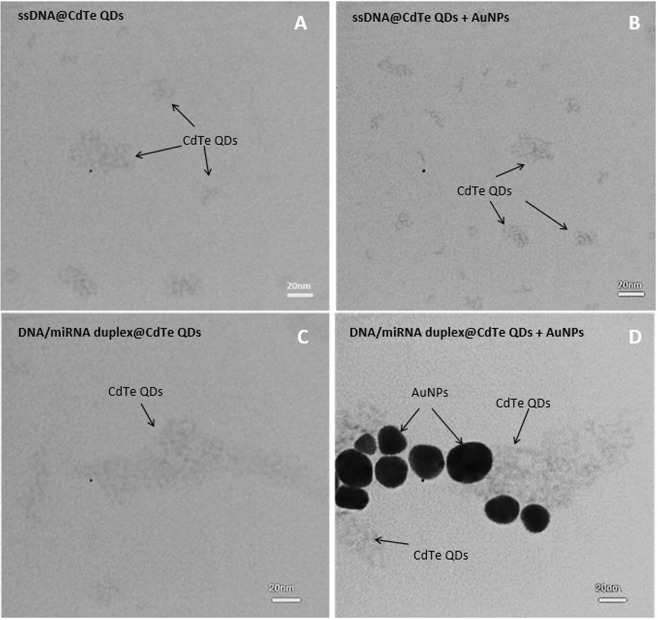
TEM images of (**A**) ssDNA@QDs, (**B**) ssDNA@QDs + AuNPs, (**C**) dsDNA/miR-155@QDs and (**D**) dsDNA/miR-155@QDs + AuNPs.

In order to illustrate the dissolution of the AuNPs by CdTe QDs photoinduction hypothesis in the absence of miRNA, TEM imaging was carried out before and after addition of the miR-155 target. Figure 5A,B are the images recorded from the sample of the DNA probe@QDs before and after addition of the AuNPs and the Fig. 5C,D after addition of miR-155 to the corresponded sample and formation of DNA/miR-155 duplex respectively. As can be seen, no AuNPs are observed in the image of Fig. 5B, while as shown in Fig. 5D, the AuNPs in the sample of containing DNA/miRNA duplex@CdTe QDs are stable.

On the other hand, comparing images of Fig. 5A,C, it can be concluded that CdTe QDs are aggregated in the Fig. 5C because they have larger dimensions than those in Fig. 5A with a super-molecular structure.

### Optimization conditions

First, quenching performance of the DNA/miRNA duplex was monitored while using various concentrations of CdTe QD. Initially, CdTe QDs with concentrations of 10 pM, 100 pM and 10 nM were used to compare the emission of free CdTe QDs, DNA@CdTe QDs and DNA/miR-155 duplex (Fig. S.2A). It can be seen that the highest quenching effect was observed at 100 pM and thus, this concentration was used in all further experiments.

To study the effect of AuNPs concentration on absorbance band intensity, various concentrations of AuNPs (0.97 nM, 1.32 nM, 1.6 nM and 1.83 nM) were investigated in the presence and absence of miRNA by comparing the absorbance band intensity of DNA@CdTe QDs and DNA/miR-155 duplex (Fig. S.2B). The figure shows that the highest difference in absorbance band intensity is obtained at 1.6 nM between DNA@CdTe QDs and DNA/miR-155 duplex, so this concentration was used for subsequent experiments.

### Colorimetric and fluorimetric sensing

**Figure 6:**
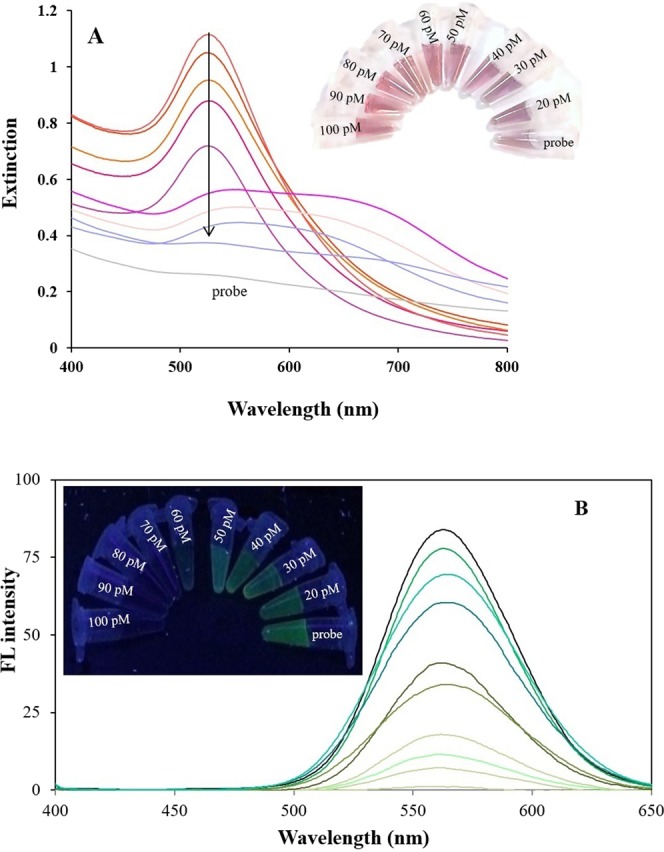
Extinction band (**A**) and emission spectra (**B**) of dsDNA-green QDs complex formed with miR-155 target in concentrations ranging 0 (probe), 20, 30, 40, 50, 60, 70, 80, 90 and 100 pM, after addition of AuNPs. (inset) Photographs of them under visible and UV light, respectively. The logarithmic plot for absorbance and fluorescence intensity against target microRNA155 concentration.

The effect of CdTe QDs on the extinction band of AuNPs is shown in Fig. 6A. In this analysis, the concentration of QDs is 100 pM, and the AuNPs has a volume of 40 μL (4.4 nM). In the absence of miR-155, the ssDNA probe@ CdTe QDs with emission at 560 nm leads to fading down of that plasmonic AuNPs extinction band at 530 nm. The effect of different concentrations of miR-155 from 20 to 100 pM on the extinction band of AuNPs is shown in Fig. 6A. Based on the results, good linear relationships were found between the absorption (A_530_) and the concentration of miR-155 in the range 20 to 100 pM, with a regression equation of A = 0.01C + 0.1268 (R^2^ = 0.962) (Fig. S3). The detection limit for miR-155 was determined to be 6.2 pM (S/N = 3). By increasing the concentration of miR-155, the extinction band intensity increases as the intensity of CdTe QDs emission decreases due to the increase in the aggregation of them and following the AuNPs plasmon excitation does not occur. Then, the reaction solution color was red. The interaction between different concentrations of DNA/miR-155 duplex and CdTe QDs affect the QDs aggregation degree and the extinction band change of the AuNPs.

Upon addition of AuNPs to DNA/miR-155 duplex mixture, QDs fluorescence emission at 560 nm was turned off, at all concentrations (Fig. S4). After completion of the reaction (after about 30 minutes), the fluorescence was recovered and decreased with increasing concentration of DNA/miR-155 duplex (Fig. 6B). As a result, a linear relationship was obtained from the plot of fluorescence intensity as function of the concentration of miR-155 over the range of 20 pM–100 pM (Fig. S5). The detection limit for miR155 was estimated to be 4.4 pM.

### To detect miR-155 in cell lysate

**Figure 7:**
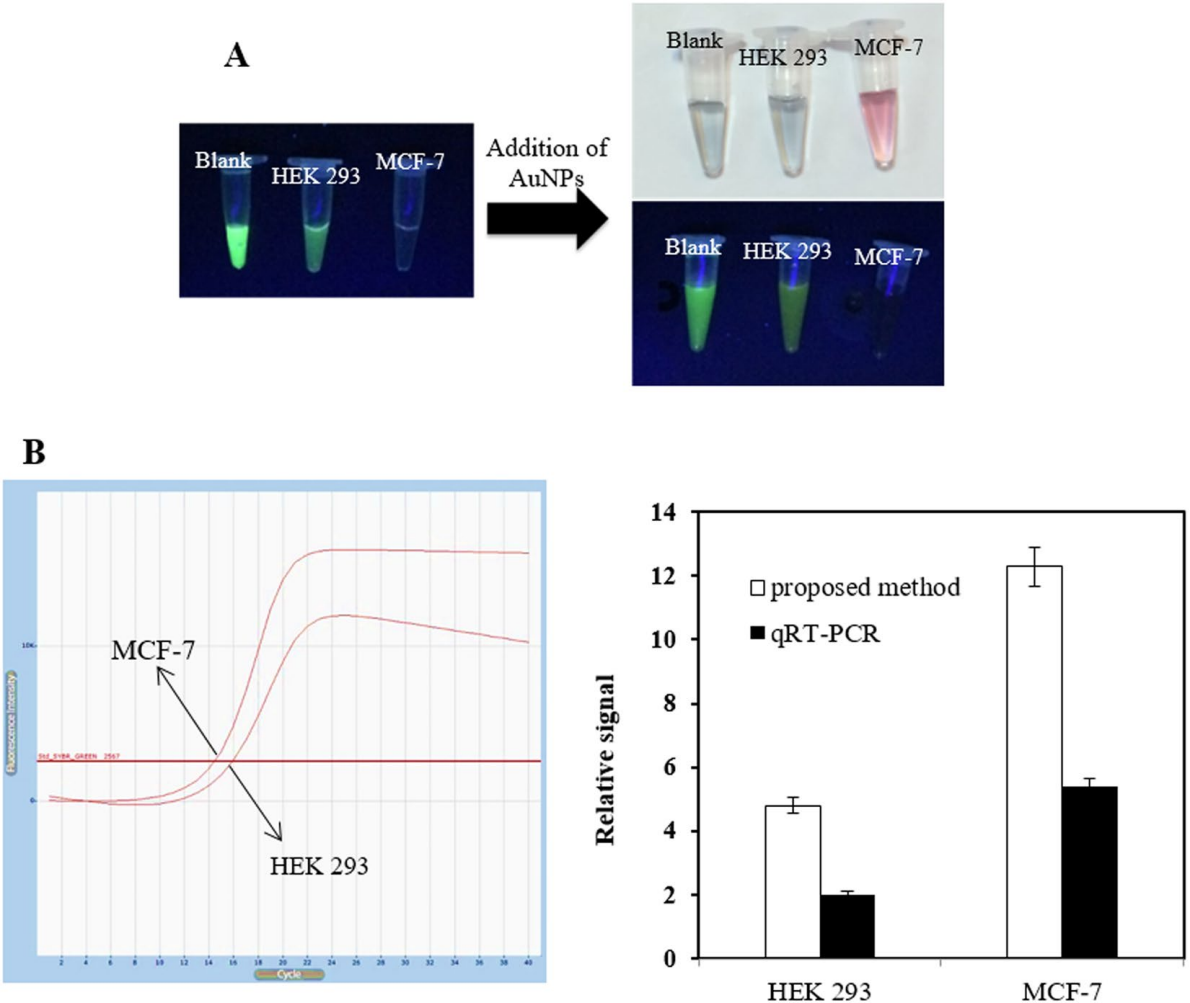
(**A**) Extinction and fluorimetric signal of RNA extracts from them and their images under visible and UV light illumination, (**B**) qRT-PCR signal of them, and (**C**) Comparison of two methods for miRNA detection. (for qRT-PCR: the relative intensity was evaluated and normalized to the expression of U6 small nuclear RNA (U6 snRNA)).

To evaluate the feasibility of this platform in real sample assays, the LSPR-based biosensor was investigated by measuring miR-155 in two dissimilar human cell lines of MCF-7 and the normal cell line of HEK 293. As shown in Fig. 7A, before addition of AuNPs, the QDs emission intensities on the test lines of the one tumor cell lines were considerably less than the human normal cell line, which was employed as the normal expression control group. In order to have a quantitative evaluation, the intensities of the test lines were estimated (see Fig. 7B). The results show the distinct miR-155 difference expression between HEK 293 and MCF-7, after addition of AuNPs. They were also in good agreement with the qRT-PCR results. In qRT-PCR analysis, U6 small nuclear RNA (snRNA) was employed as the universal endogenous control and the relative expression was calculated using the equation:

$${\rm{Fold}}\,{\rm{change}}={2}^{-{\rm{\Delta }}{\rm{\Delta }}\mathrm{Ct}}.$$

The sequences of primers for qRT-PCR are listed in Table S1.

## Conclusion

In summary, here we present a novel method towards plasmon-mediated hot-electron generation for miRNA detection, in which hot-carriers are produced proficiently at the plasmon resonance frequency of AuNPs by CdTe QDs photoinduction at about 560 nm. The resultant imposed confinement of electrons within the AuNPs in direct interaction with water, provides a new pathway for direct injection of hot electrons into the unoccupied orbitals of adsorbed water molecules.

## Supplementary information

Supplementary data
