# Supplementary material for: A New Eye Dual-readout Method for MiRNA Detection based on Dissolution of Gold nanoparticles via LSPR by CdTe QDs Photoinduction
Source: Sci Rep. 2019 Apr 1;9:5453. doi: 10.1038/s41598-019-41898-4 (PMC6443804; doi:10.1038/s41598-019-41898-4)
Supplement: Supplementary file 1 — Supplementary data [file 41598_2019_41898_MOESM1_ESM.docx]

**Supplementary data**

**A New Eye Dual-readout Method for MiRNA Detection based on Dissolution of Gold nanoparticles via LSPR by CdTe QDs Photoinduction**

**Yasaman-Sadat Borghei^1^ & Morteza Hosseini^1,2^**

*^1^Department of Life Science Engineering, Faculty of New Sciences & Technologies, University of Tehran, Tehran, Iran.*

*^2^Medical Biomaterials Research Center, Tehran University of Medical Sciences,Tehran, Iran*

*E-mail address:smhosseini@khayam.ut.ac.ir*

**Supplementary data**

**Table S1:** Oligonucleotides Sequence Used in This Work.

| Oligonucleotide name | sequence (5´ to 3´) |
| --- | --- |
| DNA probe | ACC CCT ATC ACG ATT AGC ATT AA |
| Non complementary DNA target | AAG GAA CAG TAT TAA TAG AAT GG |
| MiR-21 | UAG CUU AUC AGA CUG AUG UUG A |
| Let-7a | UGA GGU AGU AGG UUG UAU AGU U |
| MiR-155 | UUA AUG CUA AUC GUG AUA GGG GU |
| MiR-155 RT | GAA AGA AGG CGA GGA GCA GAT CGA GGA AGA AGA CGG AAG AAT GTG CGT CTC GCC TTC TTT CAC CCC TAT |
| MiR-155 Forward | GCG GTT AAT GCT AAT CGT GAT A |
| MiR-155 Reverse | CGA GGA AGA AGA CGG AAG AAT |
| U6 small RNA RT | AAA ATA TGG AAC GCT TCA CG |
| U6 small RNA Forward | CGC TTC GGC AGC ACA TAT ACT AAA ATT GGA AC |
| U6 small RNA Reverse | GCT TCA CGA ATT TGC GTG TCA TCC TTG C |


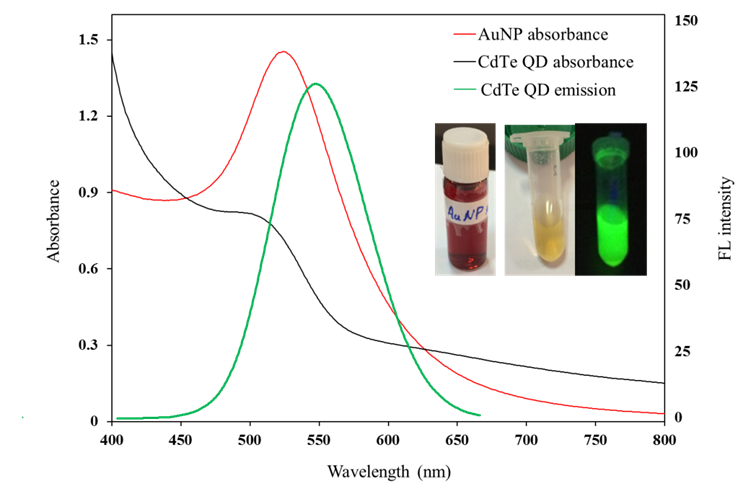


(a)

**Fig. S1:** (a) Absorbance and fluorescence spectra of AuNPs and CdTe QDs. Images of them under visible and UV light and TEM image of CdTe (b) QDs and (c) AuNPs


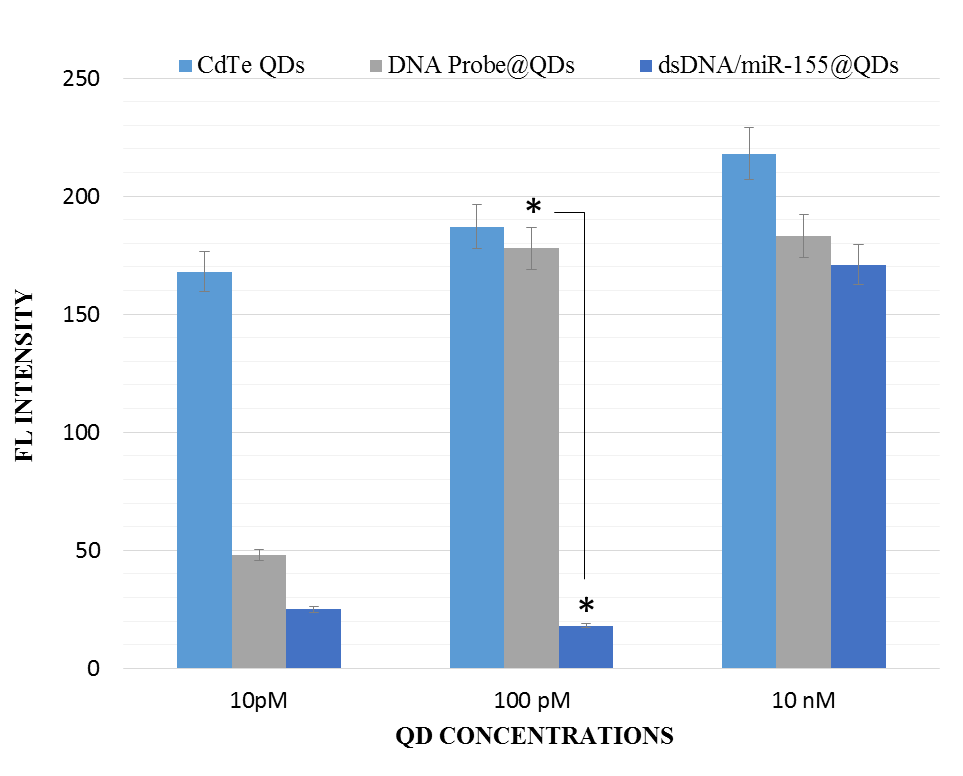


**A**


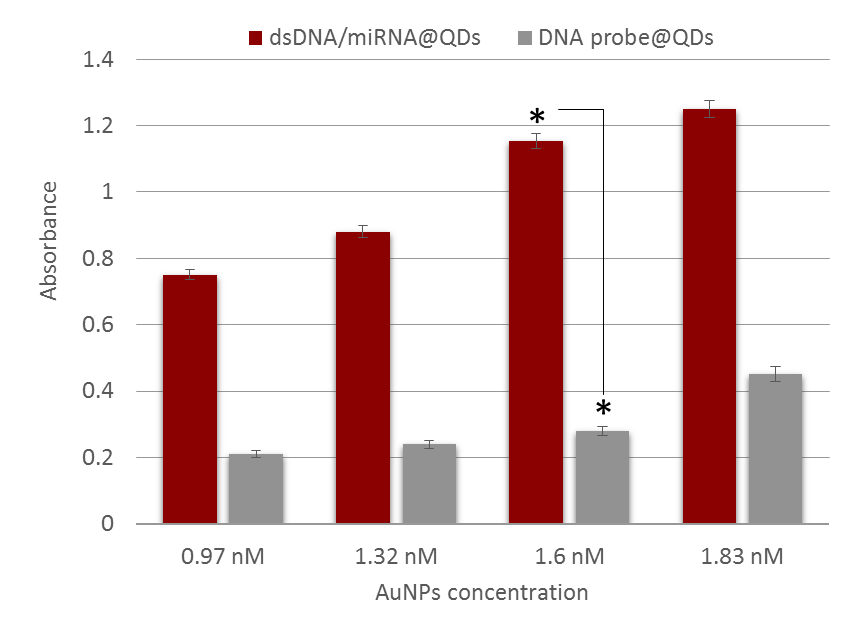


**B**

**Fig. S2:** Optimization conditions: (A) the effect of different concentrations of CdTe QD on quenching effect of the DNA/miRNA duplex on CdTe QDs, (B) the effect of different concentrations of AuNPs on absorbance band intensity in the prescence and absence of miRNA.

**Fig.S3** the calibration plot for A_530_ versus target miRNA155 concentration


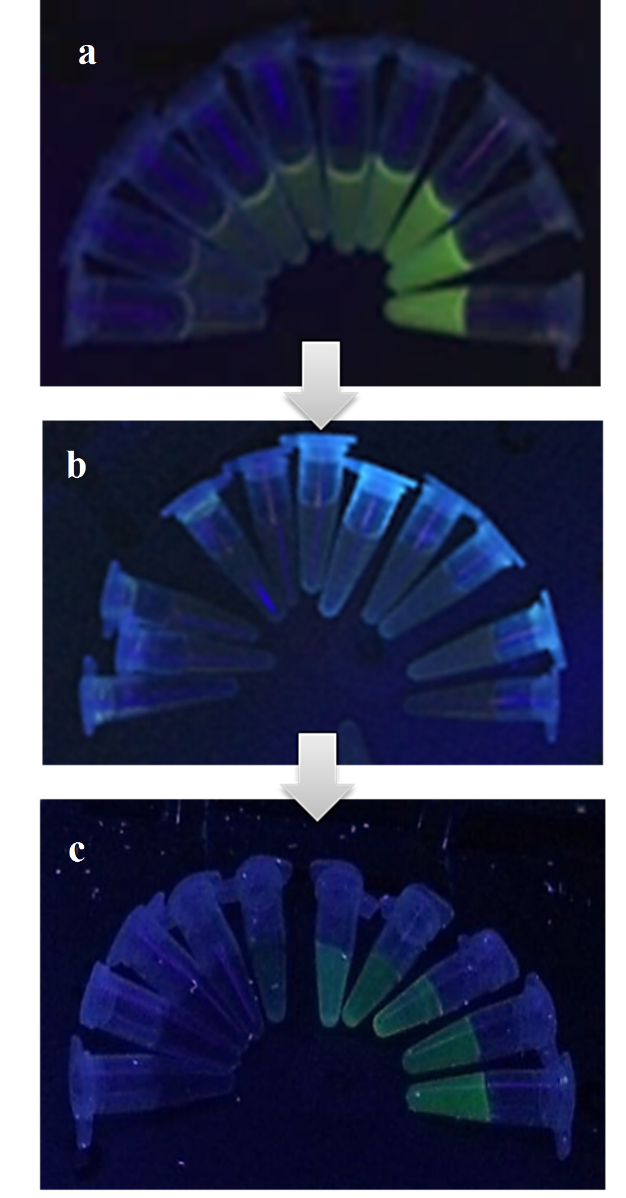


**Fig. S4:** Photographs of the fluorescent behavior of (a) dsDNA/miR-155@QDs complex, (b) dsDNA/miR-155@QDs complex + AuNPs at 0 min, (c) dsDNA/miR-155@QDs complex + AuNPs at 30 min.

**Fig.S5** calibration curves for detection of miRNA
